# The Role of Phenytoin in the Treatment of Localization Related Epilepsy: An International Internet-Based Survey of Neurologists and Epileptologists

**DOI:** 10.1155/2013/613456

**Published:** 2013-07-01

**Authors:** Rohit R. Das, David A. Griesemer, Sanjeev V. Kothare

**Affiliations:** ^1^Department of Neurology, University of Louisville, School of Medicine, Louisville, KY 40202, USA; ^2^Division of Neurology, Department of Pediatrics, Tufts University, School of Medicine, 755 Washington Street, Boston, MA 02111, USA; ^3^Department of Neurology, Children's Hospital Boston and Harvard Medical School, 300 Longwood Avenue, Boston, MA 02115, USA

## Abstract

Phenytoin (PHT) has been the most widely used medication to treat both partial and generalized seizures. However, over the past twenty years, a variety of new compounds have been released with comparable efficacy, fewer adverse effects, and more predictable pharmacokinetic properties. We surveyed neurologists and epileptologists to determine current practice patterns relating to the use of PHT using an online survey instrument. A total of 200 responses were obtained though response rates for each survey question varied. Of the respondents, 78.1% were epilepsy specialists; 60% were adult practitioners; and the remainder saw either, only children or both adults and children. For new onset partial seizures only 10 respondents said PHT would be their first or second choice, while 45% reported that they would not consider PHT. This study shows that in the era of newer medications, the role of PHT has been placed in the category of a reserve medication in intractable epilepsy.

## 1. Introduction

Phenytoin (PHT) was first synthesized in 1908 at the University of Kiel in Germany. Anticonvulsant properties of this compound were first described by Merritt and Putnam in 1938, and PHT was brought to market by Parke Davis later that year [1–3]. Since its introduction as an anticonvulsant, PHT, marketed under the trade-name Dilantin, has been the predominant medication for the treatment of epilepsy for over 7 decades. The introduction of an IV formulation and later an IV pro-drug formulation (fosphenytoin) led to this medication being used as the first choice in the treatment of status epilepticus and acute repetitive seizures. However, with the introduction of numerous newer antiepileptic medications, with fewer adverse effects, better pharmacokinetic profiles, better patient tolerability, and proven efficacy, the role of phenytoin as a treatment of choice in epilepsy has become uncertain.

The aim of the current study was to determine practice patterns among neurologists and epileptologists with regard to the use of PHT in the treatment of epilepsy. We also sought to understand the reasons why physicians prescribed (or did not prescribe) this important medication. We therefore surveyed neurologists and epileptologists to ascertain current practice regarding the use of PHT in treating persons with epilepsy.

## 2. Methods

An online survey with eleven questions was created using the website http://www.surveymonkey.com/. A list of questions in the survey (and individual response rates for each question) is found in Table 1. Questions were designed to capture demographic and clinical characteristics of respondents as well as practice patterns. A hyperlink to the survey was emailed on two successive occasions, to 1650 members of the American Epilepsy Society (AES) who received the link in the fortnightly email newsletter of the society. A total of 59 responses were obtained in this manner with a response rate of 3.5%. Subsequently, a link to the survey was emailed to 400 randomly chosen members of the AES, with membership and email contact information obtained using the AES online membership database. Members who identified themselves as neurosurgeons, pharmacists, psychiatrists, and psychologists were excluded. One hundred and thirty-nine responses were obtained (response rate: 34.75%), making for a total of 200 responses, though not all responders answered every question on the survey.

All statistical analyses were performed using the Fisher exact test to determine levels of statistical significance for dichotomous variables. Given that the study involved a survey instrument, human subjects committee approval was not required at the University of Louisville.

## 3. Results

**Figure 1:**
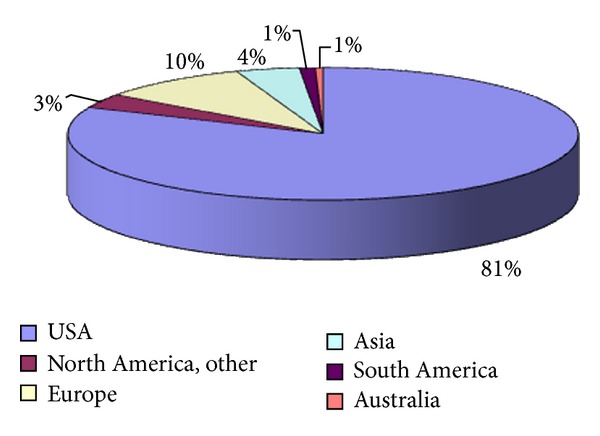
Geographical distribution of respondents (percentages).

Two hundred individuals responded to the online survey. One hundred and sixty-two described themselves as epileptologists, thirty-five as neurologists; three respondents included a pediatrician, a pharmacist, and the director of a neurophysiology school. One hundred and twenty reported primarily seeing adult patients, fifty-three saw only children, and twenty-seven reported seeing both adults and children. Four-fifth of the responses came from respondents who described their geographical area of practice as the United States; the rest of the responses were from Europe and Asia. A more detailed description of geographical distribution of respondents may be found in Figure 1.

With regard to clinical practice, virtually all respondents (*n* = 192, 97.4%) saw patients with intractable epilepsy. Sixty-one respondents reported seeing between 11–20 outpatients with epilepsy per week, 57 saw between 20 and 40 a week, 55 saw up to 10 per week while, 25 saw more than 40 patients a week in clinic. Sixty-one respondents noted that between 50%–75% of their outpatients had refractory epilepsy while 77 reported that 25%–50% of their patients had refractory epilepsy. Only 16 respondents stated that more than 75% of their outpatients had refractory seizures; 45 saw less than 25% with refractory seizures. Respondents were hesitant in their decision to initiate PHT for their outpatients with 193 respondents reporting that they started PHT for epilepsy in less than 5 outpatients, on a weekly basis. Only one respondent initiated more than 10 patients on PHT in a week. One hundred and fifty-eight respondent noted seeing up to 5 patients a week who already were on PHT, thirty saw between 6–10 per week, and ten reported seeing greater than 10 per week already on PHT.

**Figure 2:**
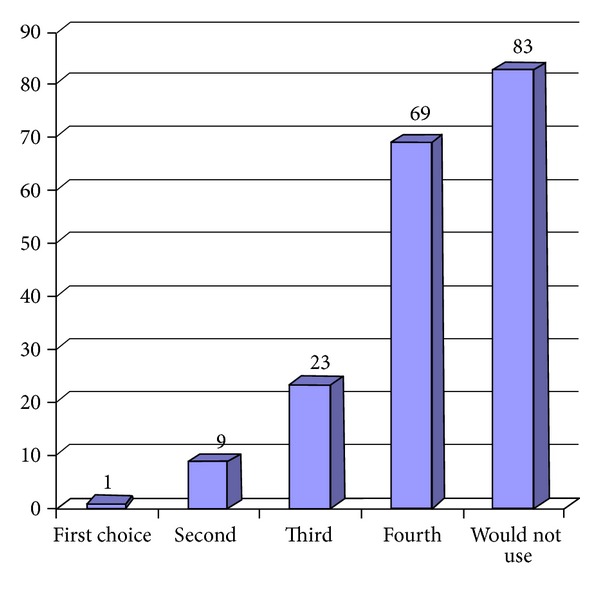
What choice is Phenytoin for a patient with new onset localization related epilepsy?

Only one respondent stated that he/she would use PHT as first choice for newly diagnosed localization related epilepsy, with 45% unwilling to even consider this medication. More information in this regard can be found in Figure 2. Interestingly, only one respondent did not think that PHT was effective in treating epilepsy with 129 stating that PHT was very effective in treating seizures and 69 stated that PHT was somewhat effective. Thirty-seven respondents reported that up to 20 patients a year discontinued PHT, 46 reported that greater than 20 patients a year stopped PHT, all on account of side effects.

Two respondents, both pediatric neurologists, individually emailed us their unsolicited opinions about the role of PHT in current clinical practice. One pointed out that (there is) “virtually no role for maintenance phenytoin in pediatric epilepsy management. I have been practicing for 25 years and cannot remember using it except for a very short time in early post-traumatic seizures [in severe head injury].” The second respondent opined that “most ED physicians, primary care docs (sic), and patient families do not understand zero-order kinetics and the need to make changes in small increments. Consequently, the kids on phenytoin bounce in and out of EDs with toxicity or breakthrough seizures.” The respondent, however, felt that there was a special role for PHT in treating seizures that arise in the frontal lobe.

To improve statistical power for testing of significance, we collapsed respondent choices of PHT of first to fourth choice into one group and those who would not use PHT into a second group. Using Fischer's exact test, we compared respondents' choices for epilepsy treatment between those who treated adults with epilepsy as compared to those who treated children or children and adults. Fifty-eight percent of adult practitioners reported that PHT was among their first four choices for initial therapy of localization related epilepsy as compared to 43% of pediatric practitioners. The trend was nonsignificant (*P* = 0.1). Similarly, we also examined differences in choosing PHT as a treatment for localization epilepsy between physicians who saw only adult patients with epilepsy on the one hand (*n* = 110) and those who saw children exclusively and those who saw both adults and children on the other hand (*n* = 80). The difference again was nonsignificant (*P* = 0.38). Finally, we again examined difference between those who practiced in the United States (*n* = 162) as compared to those who practiced elsewhere in the world (*n* = 37); the difference was again nonsignificant (*P* = 0.59).

## 4. Discussion

Prior to the introduction of PHT in 1938, the principal pharmacological mechanisms of treating seizures were phenobarbital, borax, and the bromides. By 1940, PHT became the most important and frequently used drug to treat epilepsy as noted by the Annual Review of Epilepsy published that year [4]. PHT has been the first choice drug in the treatment of epilepsy since then, though the introduction of carbamazepine (CBZ) and valproate (VPA) in the 1970s led to these drugs becoming first choice medications for partial onset (CBZ) and childhood epilepsies (VPA) [5]. Between 1989 and 1994, five new anticonvulsants were introduced: felbamate, vigabatrin, gabapentin, lamotrigine, and oxcarbazepine. Between 1994 and 2009 a further nine new medications came to market; the most important of which are topiramate and levetiracetam [5]. Of these medications, oxcarbazepine, lamotrigine, levetiracetam, and topiramate have become very important choices for clinicians when prescribing anticonvulsants in large part due to favorable pharmacokinetics, fewer adverse events, and better patient tolerability.

Very few studies have examined current anticonvulsant prescribing trends by clinicians. Using a large American database of patients with a new diagnosis of epilepsy, based on Medicare and Veterans Affairs data, Hope et al. found that PHT was the initial AED in two thirds of all patients, in the years between 1999 and 2004 [6]. In Australia, Hollingworth and Eadie found that, despite the introduction of a number of newer medications, the most commonly used medications, between 2002 and 2007, were PHT, CBZ, and VPA with the latter being the most popular [7]. In Taiwan, in 2004, PHT was the second most popular medication after CBZ for epilepsy monotherapy [8]. In Italy, prior to 2005, phenobarbital, carbamazepine, and phenytoin were the major AEDs used in the treatment of epilepsy. Following 2004, gabapentin was reported, in a large prescribing database study, to be the most popular AED. The authors of this study report that in more recent years, newer AEDs have become more frequently used in older patients [9]. A regional Italian study reported a 25% increase in prescriptions for newer AEDs between 2004 and 2005 [10].

Occasional surveys have examined the issue of physician choices of anti convulsants. Burneo and McLachlan surveyed Canadian neurologists, asking what medication they would use given a variety of clinical situations. VPA was the first choice for generalized epilepsy in males, lamotrigine for females. CBZ was the first choice for the treatment of localization related epilepsy, lorazepam for status epilepticus [11]. In a survey of opinion leaders in epilepsy, Karceski et al. found that PHT was not a first choice medication in the treatment in any seizure type in adolescents and adults. The authors did not however seek an opinion on the first choice in treating status epilepticus [12]. A French survey found that PHT was not in the top three and most often top five choice of epileptologists for a range of epileptic syndromes, from absence seizures to localization related epilepsy [13].

Our study is the first to examine the opinions of neurologists and epileptologists with regard to the continued use of PHT in treating epilepsy. The majority of respondents in the survey were from the United States, which is not surprising given that the survey was distributed through the American Epilepsy Society. However, respondents practiced in every continent excluding Africa. A slight majority were adult practitioners, with 40% seeing either children alone or both adults and children; almost all respondents received referrals for the evaluation of intractable epilepsy. The majority of respondents saw between 20–40 patients with epilepsy on a weekly basis. A significant number of these patients had intractable epilepsy.

Forty-five percent of respondents stated that they would not consider using PHT in a patient with newly diagnosed localization related epilepsy. Only one respondent was willing to use PHT as a first choice medication for this condition. This study shows that the vast majority of respondents believe that PHT is effective in treating seizures. This is in line with evidence based guidelines which have demonstrated that the highest level of evidence exists to support the use of PHT as initial monotherapy for adults with localization related epilepsy [14]. However, virtually all respondents had seen at least 10 patients annually who had had adverse events related to PHT that lead to discontinuation of this AED. The vast majority also reported initiating only a small number of patients with newly diagnosed localization related epilepsy with PHT therapy.

Very importantly our survey found no significant differences across a variety of different physician groups regarding prescribing choices using PHT for new onset localization related epilepsy. Historically, PHT has been more important in treating epilepsy in adults, but in this study, no significant differences emerged between physicians who saw adults only and those who saw children. Again, no differences emerged between neurologists and epileptologists in this regard. Finally, physician opinions did not differ between those who practiced in the United States and those outside the US. This is very important, as in many nations with socialized systems of health care, the primary focus remains on reducing costs and as an older medication with many generic forms, PHT is relatively inexpensive.

The number of PHT side effects, both acute and chronic, the narrow therapeutic index, and poor patient tolerability compare poorly with newer medications like levetiracetam and lamotrigine which have much fewer side effects and a broad therapeutic index. There may still remain an important role for PHT in the treatment of status epilepticus with a recent European treatment guideline recommending IV PHT as treatment of choice for convulsive and nonconvulsive SE that does not respond to lorazepam; this recommendation was supported by high quality evidence [14]. We did not examine clinician opinions regarding treatment of SE with PHT in this survey.

As in any observation made based on surveys, our findings need to be interpreted in the context of low recruitment of physicians completing the survey, and the intrinsic bias that exists when summarizing findings from surveys. Nevertheless, our survey therefore demonstrates that while PHT is an excellent medication for controlling seizures, it is no longer a frontline medication in treating epilepsy due to several other important factors. The role of PHT most likely will be that of a reserve medication for intractable epilepsy.

## Figures and Tables

**Table 1:** Survey questions and response rates.

Questions	Number of responses
*Demographic information questions *	
Is your primary specialty neurology or epilepsy?	200
Do you see adult patients, pediatric patients, or both?	200
What best describes the geographical location of your clinical practice?	199
*Clinical practice information questions *	
Do you receive referrals for the management and further evaluation of intractable epilepsy?	198
On average, over the course of a week, how many patients with epilepsy do you see, as outpatients?	198
What percentage of your patients have refractory epilepsy?	199
*Questions pertaining to use of phenytoin *	
On average, in how many patients, per week, do you initiate phenytoin as anticonvulsant therapy?	194
How many patients do you see on a regular basis that are already on phenytoin anticonvulsant therapy?	198
In your opinion, what choice is phenytoin for a patient with new onset partial seizures?	185
On average, how many patients have you seen with side effects from phenytoin that have led to the discontinuation of the medication?	188
In your opinion, how effective is phenytoin in controlling seizures?	199
